# Are social networks effective in promoting healthy behaviors? A systematic review of evaluations of public health campaigns broadcast on Twitter

**DOI:** 10.3389/fpubh.2022.1045645

**Published:** 2022-11-22

**Authors:** Mireia Faus, Francisco Alonso, Arash Javadinejad, Sergio A. Useche

**Affiliations:** ^1^Faculty of Psychology, University of Valencia, Valencia, Spain; ^2^INTRAS (Research Institute on Traffic and Road Safety), University of Valencia, Valencia, Spain

**Keywords:** public health, communication campaigns, social marketing, evaluation, Twitter, social media

## Abstract

**Introduction:**

While public health campaigns disseminated through Twitter have multiple theoretical advantages over other strategies (e.g., a high potential reach and low economic cost), the effectiveness of social networks as facilitators of attitudinal and behavioral changes in the population seems to remain weakly supported. Therefore, this systematic review was aimed to analyze the degree of impact of healthy behavior-related campaigns as documented in scientific literature.

**Methods:**

Strictly following the PRISMA methodology, a total of 109 indexed articles were obtained, of which only 18 articles met the inclusion criteria. In addition to accessing the literature available on WOS, Scopus, BVS, Medline, Cochrane Library and PubMed, the quality of the existing studies was assessed through the Critical Appraisal Skills Programme (CASP) protocol.

**Results:**

The results of this systematic review revealed a small number of evaluations of the effectiveness of social campaigns disseminated on Twitter, although the quality of these studies was considerably good. Most of the research used statistics and metrics for evaluation, with residual use of other measurement methodologies. However, their effectiveness and impact on public health-related behaviors remain arguable, in view of the existence of marked tendencies to: (i) not evaluate these campaigns; (ii) evaluate them through excessively brief, ambiguous, or potentially biased indicators; and (iii) not carry out systematic follow-ups over time.

**Discussion:**

Although there is no strong evidence of the suitability of Twitter as a suitable medium for raising public health awareness on behavioral health affairs, the actual limitations identified in this review would help to optimize this paradigm and enhance the quality, reach, and effectiveness of such communication strategies.

## Introduction

Typically, social marketing strategies tend to use very generic marketing practices (i.e., those used for advertising a variety of goods) in the attempt to raise public awareness of a wide range of issues (1), partly as a result of the assumption that behavioral changes might uniformly act at different levels, and for several purposes (2, 3). Indeed, recent macro-social critical events such as the COVID-19 pandemic have facilitated that, given their high accessibility and coverage, social networks have been systematically used to divulgate campaigns, programs or interventions on public health under approaches compatible with the basic principles of social marketing (4, 5). Social networks are dedicated websites or applications that allow users to communicate with each other by facilitating the exchange of information through the posting of messages, comments and images. In these cases, campaigns are usually aimed at preventing negative behaviorally-influenced outcomes affecting public health, such as road crashes, air pollution or viral contagions (6–8). Complementarily, there are also campaigns specifically aimed at people who present a certain health problem, and their families, with the purpose of creating support groups or promoting tools that might enhance their quality of life (9).

Like any other publicity campaign, cost-effectiveness becomes a critical factor in determining the effectiveness of campaigns in raising awareness among the audience (10). In this regard, scientific evidence is somewhat inconsistent. For instance, while several studies suggest that broadcasting advertising campaigns on television remains something effective to achieve behavioral change in citizens, others remark what they call “a clear advantage” of digital channels nowadays (11, 12). Indeed, a comparative study performed by Allom et al. (13) shows that campaign broadcast solely through digital media was significantly more cost-effective than the same campaign broadcast on television, taking into account not only the economic factors but also the impact on individual behavior. This finding is consistent with other research indicating that online broadcasting of campaigns to reduce tobacco consumption was more beneficial than the joint presentation of the spot in digital media, press and radio (14).

In the same line, some other applied researches have shown that, while product advertising usually achieves the expected effectiveness, social campaigns through traditional media fail to modify behaviors in favor of healthier behaviors and lifestyle habits (15). However, and especially as the current coverage of social media remains low in some age and income segments, it seems unlikely that traditional media, and particularly television, would cease to be the main broadcast channels for social advertising (16). This phenomenon is due to the fact that they continue to be an important source of entertainment and information for many people, with a high degree of reach spanning all social classes (17).

### Twitter as a communication channel with the public

The good results obtained by digital media in many terms have contributed to “open the door” to a new line (technical term) for developing public health campaigns. During recent years, social networks have been a way to raise awareness of different issues (18). In particular, Twitter is a low-cost channel with a potentially high reach and impact, something in fact evidenced by its high revenue, currently estimated in over 5 billion dollars. Moreover, it presents a mostly young audience, which prefers to be informed on social networks rather than traditional media (19). Therefore, developing awareness campaigns on Twitter either as the sole broadcast medium -or as a potential complement to campaigns launched by other media- could be particularly beneficial for reaching key audiences in public health terms, such as young people, at-risk minorities and other individuals of interest in terms of public health (20).

The latest official data indicate that worldwide 500 million tweets are posted every day (21). These figures offer the possibility that millions of people can receive a campaign message without geographical restriction. However, at the same time, the large amount of content makes it difficult for the message to stand out from the rest, and for the audience to reflect on the issue addressed (22). Therefore, assessing the impact of communication campaigns transmitted by Twitter would add important insights to further analyze (e.g.) which factors capture the attention of users, and which ones influence recall and attitude change.

### Objectives of the study

The core aim of this systematic review was to analyze the reported impact and effectiveness of behaviorally-based public health campaigns broadcast on Twitter. Additionally, we applied assessment tools commonly used for this type of campaign, in order to detect the possible methodological limitations of the methodologies used to assess such issues on communication campaigns.

## Methods

Overall, systematic literature reviews consist of a comprehensive mapping of scientific evidence based on a protocol and a transparent and systematic methodology to find and explain in detail a research question (23). The present review was conducted according to the quality standards and protocol established by PRISMA, last updated in 2020 (24), and following the recommendations of the Cochrane Review Group (25). All the four authors of the article acted as independent reviewers, each performing the selection, evaluation and data extraction of the studies. Discrepancies that arose were resolved through consensus decisions.

Five steps were followed in the development of the systematic review:

(1) Identifying the research question.
(2) Finding relevant studies.
(3) Selecting the studies.
(4) Charting and collating the data.
(5) Summarizing, analyzing and reporting the results.

### Step 1: Identifying the research question

The aim of this systematic review was to determine the degree to which healthy behavior-targeted campaigns broadcast on Twitter are reported as effective in their evaluation studies. Consequently, this study seeks to identify the number and category of scientific studies that have evaluated the efficacy of a health campaign carried out through this social network.

In this sense, articles that analyze the opinion of Twitter users regarding social or health issues were discarded. Neither there were be taken into account those investigations that collect reactions to campaigns issued through other media or any other type of study in which the objectives are not to evaluate the effectiveness or impact of a campaign published using Twitter. No comparisons were made between the studies. The results include a summary and a thematic analysis of all the selected articles.

### Step 2: Finding relevant studies

The present investigation was conducted following the PRISMA 2020 guidelines for reporting systematic reviews (24). In the first instance, a scoping review (a standard literature search, as performed in the case of empirical papers) of the literature was performed to preliminarily assess the potential and scope of the research objectives. In addition, it served to identify key terms that would be applied in the searches for the next phase of the systematic review process.

Subsequently, six databases were used for the preliminary literature search, which were selected because of their recognition as reliable quality indicators valued by the scientific community. The selected databases were Web of Science, Scopus, Virtual Health Library, Medline, Cochrane Library and PubMed. We also reviewed other reference lists of different primary research scoping reviews, potentially eligible and not captured by our search strategies.

The search was conducted in the first week of August 2022. It did not have exclusion criteria related to the year of publication. Therefore, all literature published from the beginning of the database until the search date was taken into account for the present review.

The search strategy was carried out taking into account that the review covered research published in both English and Spanish. Therefore, the same Boolean search operator “(evaluation OR evaluación) AND twitter AND (health OR salud) AND (campaign OR campaña)” was used in all databases. The terms selected and the search operator were agreed upon by the authors following the information acquired during the scoping review.

### Step 3: Selecting the studies

During this step, articles that did not address our research objective (i.e., focusing on healthy behaviors and also having been broadcast on Twitter) were excluded. Given their substantial differences in terms of targets, dynamics and population quotas, no other potential social networks were considered as suitable channels for the campaigns analyzed. Therefore, all studies assessing campaigns broadcast on e.g., Instagram, Facebook and TikTok, or overlapping Twitter with other platforms were automatically excluded. Although it clearly reduces the scope of the review, it also prevents the data analysis to get affected by many biases and virtually uncontrollable effects. All authors initially, and independently, evaluated a subset of titles and abstracts and then met to discuss and resolve any discrepancies.

Only scientific articles were included, avoiding the inclusion of gray literature. Therefore, we did not select publications in the form of letters, doctoral dissertations, conferences/abstracts, editorials, case reports, protocols, or case series. We also restricted our eligibility criteria to articles published in English and Spanish to which we could obtain access to the full paper, either because they were available due to their open access status, or because they could be requested through the library system used.

### Step 4: Charting and collating the data

The data sources (papers) meeting the inclusion criteria were critically reported and analyzed using the Arksey and O'Malley's (26) descriptive-analytic method, which provides a suitable and considerably standardized set of sections to be included in the data extraction form, contained in Table 1. For each eligible paper included, the following data were extracted and recorded: author(s), year of publication, country of study, topic, brief description of the campaign, evaluation method, results (main outcomes) and key limitations.

**Table 1:** Summary of the general characteristics of the selected studies.

**References**	**Country**	**Topic**	**Campaign**	**Method of evaluation**	**Results (main outcomes)**	**Key limitations**
Schlichthorst et al. (27)	Australia	Suicide	“Man Up” campaign, which links masculinity and suicide (hashtags #MANUP, #ABCMANUP, #LISTENUP and #SPEAKUP)	Twitter statistics (followers, likes, retweets and impressions metrics)	Hashtags grew substantially during the campaign broadcast. The most frequent content was related to help-seeking, masculinity and expression of emotions. Very effective in disseminating information and promoting real-time conversations	Metrics Tweet screening Biased sample
Harding et al. (28)	Ghana	Breastfeeding	Breastfeed4Ghana Campaign	Online cross-sectional survey (*n* = 451)	Acceptability was high but 61% of the audience did not remember the purpose of the campaign. Exposure was not associated with increased breastfeeding awareness	Metrics Survey limitations
Castillo et al. (29)	Canada	Dementia	Dissemination of digital content on pain in dementia, with the hashtag ##SeePainMoreClearly	Twitter statistics (metrics and impressions)	Hashtag received more than 5,000,000 impressions and was used in 31 countries. There was a greater number of posts on the topic during the campaign broadcast period	Metrics Lack of post-test
Grantham et al. (30)	Canada	Nutrition	Campaign of a dietician for 16 weeks, with the hashtag #eatwellcovid19	Twitter statistics (metrics and follower testimonials)	Two types of followers: those who appreciated listening to stories submitted by followers, and those who appreciated evidence-based information	Metrics Campaign design
Moukarzel et al. (31)	World	Breastfeeding	World Breastfeeding Week 2020 (WBW) Campaign	Social network analysis (users and topics of conversation)	Increased conversation during the campaign. Formation of identifiable communities based on geolocation, interests and profession. Identification of influencers as a “bridge” between the public and the scientific community	Lack of behavioral assessment
Viguria et al. (32)	Spain	Eating disorders	Eating Disorder Awareness Week and Wake Up Weight Watchers campaigns, through #wakeupweightwatchers, #eatingdisorderawarenessweek, #eatingdisorderawareness, and #EDAW	Twitter statistics (impressions of collected and sorted tweets)	During the campaign there were more tweets about the topic, comparing the official hashtags with the control hashtag, which is used throughout the year (#eatingdisorder). Medical and awareness content was low. A large percentage of tweets did not promote preventive or help-seeking behaviors	Biased sample Lack of behavioral assessment
Sundstrom et al. (33)	United States	Vaccination	Campaign aimed at parents, to raise awareness about the human papillomavirus (HPV) vaccine	Twitter statistics (metrics and impressions)	More than 370,000 total impressions were reached, with pro- and anti-vaccine comments using personal experiences. Comments with misinformation were responded to and corrected by the users themselves	Not generalizable
Lenoir et al. (34)	United Kingdom	Cancer	Campaign #SmearForSmear to encourage women to take a selfie showing their lipstick going over the edge and post it, to raise awareness of cervical cancer	Twitter statistics, coding of tweets by topic and analysis of the content of the messages	More than half of the users posted the required photo, and almost a third of the tweets were awareness-raising. The awareness messages were linked to the factors “female gender”, “women who experienced an abnormal smear test” and “UK inhabitants”	Data biases Lack of behavioral assessment
Lee et al. (35)	Korea	Cancer	Korean Society of Coloproctology colon cancer campaign	Twitter statistics for the keywords “colorectal cancer,” “colorectal cancer awareness campaign,” “gold ribbon,” and/or “love handle"	The majority of the content of the tweets analyzed was spam, with only 12.6% of the messages sharing information. The impact of the campaign among Twitter users was questionable	Small sample size Data biases
Booth et al. (36)	Canada	Mental Health	Bell Let's Talk campaign on mental health awareness and utilization of available preventive services	Record of monthly mental health visits in Ontario outpatient clinics	Twitter inclusion in the campaign was associated with increased utilization of mental health and psychiatric services. Especially significant was the increase in adolescents aged 10–17 years	Data limitations
Wittmeier et al. (37)	Canada	Hirschsprung's Disease	Shit Happens campaign to engage family members affected by the disease	Twitter statistics (metrics and reach)	Assessment of responsiveness showed that within 2 h of posting, a question could receive 143 views and 20 responses, increasing to 30 responses after 5 h	Biased sample Not representative
Harding et al. (38)	Ghana	Breastfeeding	Campaign to promote safe breastfeeding	Twitter statistics (metrics and impressions)	At the start of the campaign, the materials received an average exposure of 60 users. Reach on Twitter was not significant, while it was on Facebook.	Data limitations Small sample size
Gough et al. (39)	United Kingdom	Cancer	Dissemination of messages on the effects of sunlight and the prevention of skin cancer	Pre- and post-intervention household survey; Twitter statistics (metrics and reach), and coding of tweets by topic	There were a total of 417,678 tweet impressions. Shocking messages generated the most impressions, while humorous messages generated the most engagement. The survey revealed an increase in skin cancer awareness, and a change in attitudes about UV rays and tanning	Not representative Survey limitations
Ayers et al. (40)	United States	Tobacco	Great American Smokeout campaign to encourage smoking cessation	Twitter statistics (metrics and impressions) using a quasi-experimental design	There was a 28% increase in tweets related to the topic compared to the rest of the year	Metrics
Jawad et al. (41)	United States	Tobacco	ShishAware campaign warning of the dangers of pipe smoking	Twitter statistics (metrics and impressions)	Twitter enabled the most organization-based contact, but Facebook was the most interactive medium. There is no data on the effects on awareness, knowledge and attitude of users	Data limitations Lack of behavioral assessment
Friedman et al. (42)	United States	STDs	GYT: Get Yourself Tested campaign to reduce stigma and promote communication and testing for sexually transmitted diseases (STDs)	Twitter statistics and affiliate data for Planned Parenthood and infertility prevention clinics	It is estimated that the campaign reached over 52,000 youth. Subsequent years saw a 71% increase in STD testing, although cases of positivity remained stable	Data limitations
Fung et al. (43)	China	Hand washing	Global Handwashing Day Campaign	Qualitative content analysis of messages	Social networks serve as amplifiers of content provided by traditional media	Data limitations
Chung (44)	United States	Tobacco	Tips From Former Smokers is a smoking cessation campaign from the Centers for Disease Control and Prevention (CDC)	Twitter statistics (metrics and impressions)	The role of non-profit entities in disseminating the message launched by government authorities is noted. Two-way interactions with users were minimal	Data limitations Lack of behavioral assessment

### Step 5: Summarizing, analyzing and reporting the results

The data extraction is recorded in tabular form, in order to help readers to identify the key sections, features and contents of these sources. Once their relevant features and main findings were summarized, the quality of the papers included in the systematic review process were assessed through the Critical Appraisal Skills Programme (CASP) tool, whose core utility is performing a quality assessment of the studies analyzed, in order to ensure that the results are not significantly altered or biased by potential technical shortcomings present in these sources.

## Results

### Search results

**Figure 1:**
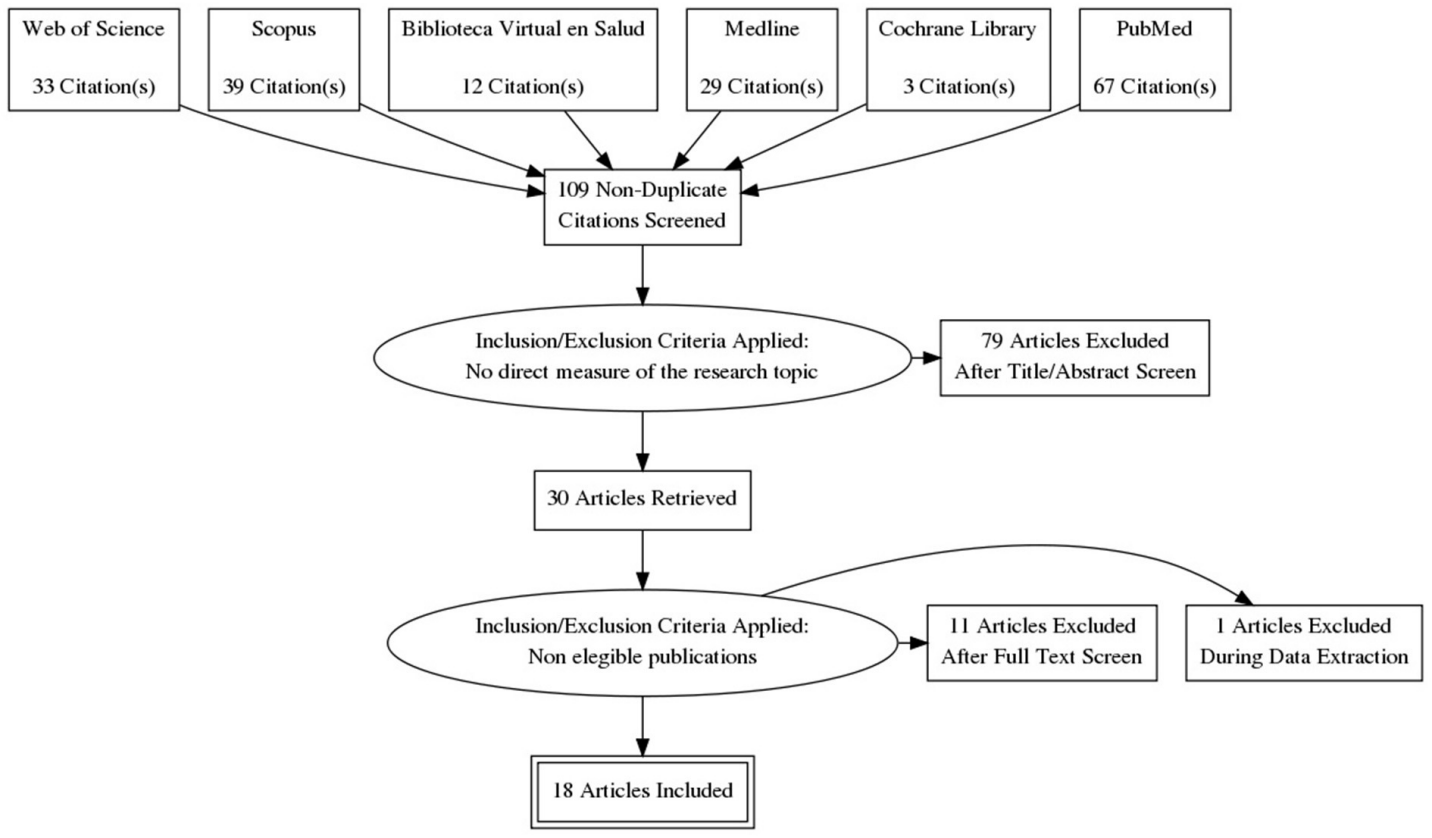
PRISMA diagram for this systematic review.

By means of the search strategy, a total of 109 possible articles were obtained for analysis, after discarding all duplicate documents. After reading the title and abstract and being preliminarily assessed by reviewers, 79 articles were discarded because they did not respond to the objectives of the review. Subsequently, a new manual screening was performed after reading the full text of the remaining articles. After this process, 18 eligible articles were obtained and included in the study. Figure 1 shows the process of searching and selecting data sources.

### Characteristics of eligible research articles

**Figure 2:**
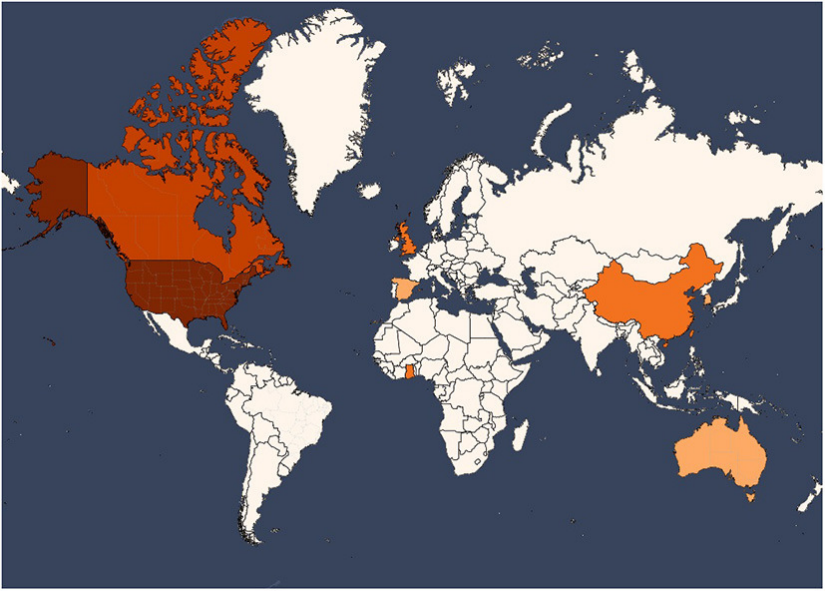
Geographical distribution (country of origin) of the selected studies.

Although the search was conducted for articles published in both Spanish and English, the 18 eligible articles were all published in English. The selected papers were published between 2014 and 2021, which is in accordance with the recentness and novelty of the subject matter of the study. In addition, the studies were conducted in geographically different countries (Figure 2). In this sense, there is representation from 8 countries located in five continents: United States (*n* = 5), Canada (*n* = 4), Ghana (*n* = 2), United Kingdom (*n* = 2), Australia (*n* = 1), Spain (*n* = 1), Korea (*n* = 1) and China (*n* = 1). In addition, one of the studies was conducted worldwide without restricting the data geographically.

Table 1 details the main characteristics and findings of the selected scientific articles.

**Figure 3:**
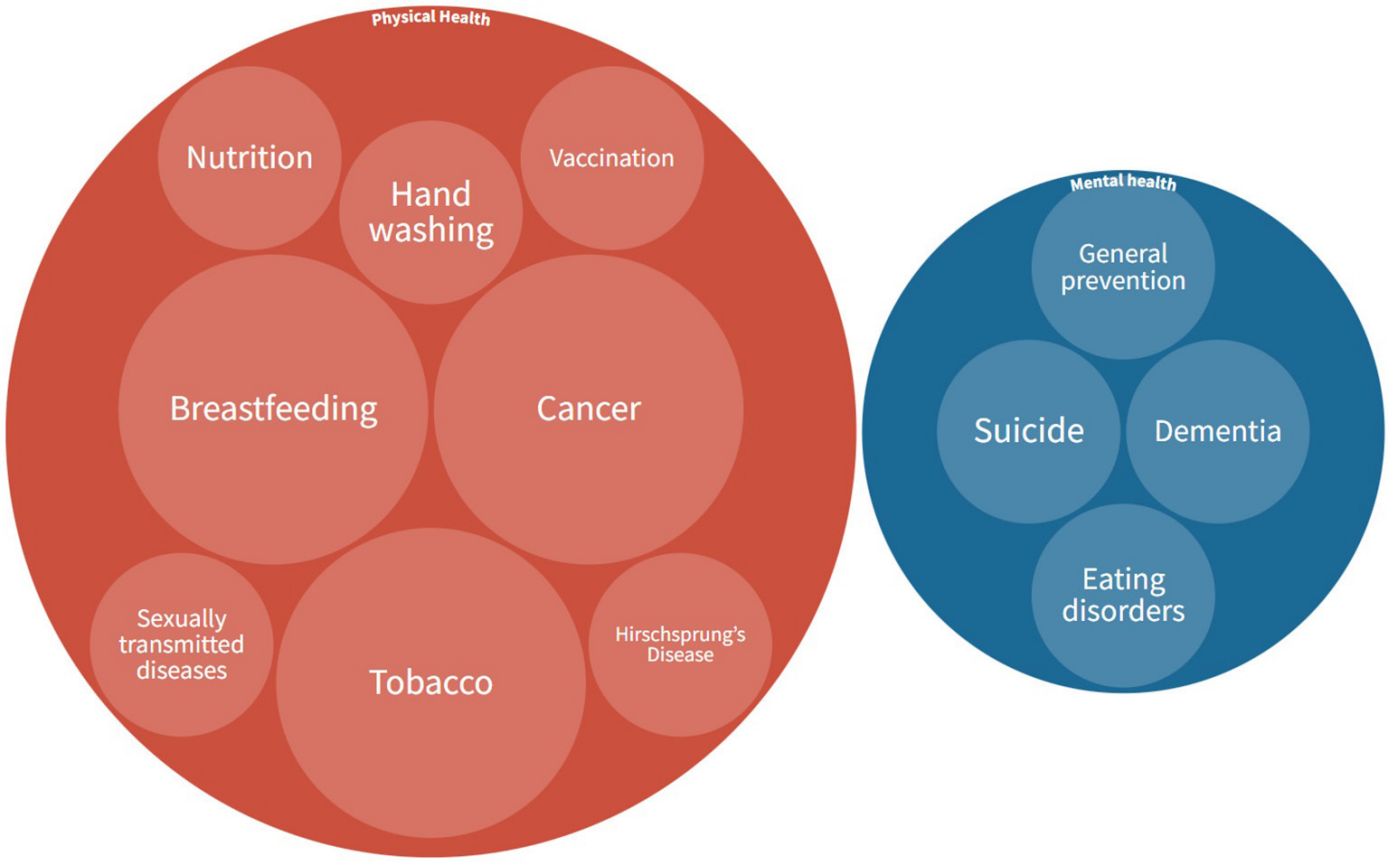
Distribution of the themes addressed by the health campaigns evaluated in the selected articles.

The evaluated health campaigns broadcast on Twitter cover a wide range of issues. Figure 3 shows the distribution of the topics of the campaigns. The data show that more physical health campaigns are evaluated (*n* = 14) than mental health campaigns (*n* = 4). Specifically, the issues that have been most emphasized are cancer prevention (*n* = 3), each of which deals with a different type of cancer, reduction of tobacco consumption (*n* = 3) and breastfeeding recommendations (*n* = 3). On the other hand, most of the health campaigns are of a preventive nature (*n* = 16), with very few referring to the treatment of diseases that are already present or manifest (*n* = 2). This is the case of the campaign on dementia and on Hirsch-Sprung's Disease, in which its broadcast *via* Twitter was not only aimed at information and awareness of the disease, but also intended to facilitate contact between people or families who were living the same situation for the formation of support groups in the distance.

The record of findings on the effectiveness evidenced by the campaigns indicates that the conversation on Twitter about the issue addressed increased substantially during their broadcast period (27, 29–31, 40, 43). In this regard, in most cases, the evaluation tools were Twitter's own statistics on metrics, impressions and reach, all measured by the numbers of followers, likes, retweets, interactions and conversations generated with the official hashtag (*n* = 16; 88.9%). Therefore, these articles generally conclude that the campaign was useful in raising user awareness.

However, it should be noted that some research uses category analysis or additional qualitative analysis that provides further information (*n* = 6; 33.3%). In some of these cases, the results of this qualitative analysis evidence that part of the users' messages was not linked to the promotion of healthy behaviors or attitudes (32, 35). Thus, it is evident that the information transmitted through Twitter fails to raise awareness among the audience. Furthermore, in the two articles comparing the impact on various social networks, Facebook is the channel that generates the most interactions, while Twitter has a residual impact (38, 41).

Few studies have conducted evaluations beyond the analysis of metrics and the social network users' own messages. Only three studies employ other instruments, and these provide somewhat contradictory data. On the one hand, Harding et al. (28) conducted a survey after the broadcast of the breastfeeding campaign in Ghana. The data collected stated that respondents remembered the campaign itself, but not its message or purpose. In contrast, the survey conducted by Gough et al. (39) revealed an increase in skin cancer awareness, and even a change in attitudes related to the intention to engage in preventive behaviors regarding this issue. Likewise, research by Booth et al. (36) and Friedman et al. (42) relied on records from clinics and outpatient centers, which evidenced a substantial increase in young people attending for treatment or prevention of the problems that the campaigns raised awareness about.

The discrepancies observed in the results of the studies may be due to limitations in the recording of information or the instruments used for evaluation (40). In this sense, a large portion of the articles in which Twitter tools were used to analyze campaign metrics and impressions specify that errors or limitations may have occurred in the data that distort the results (n = 11; 61.1%). In addition, an important limitation is that, except in two of the investigations, no post-campaign follow-up was performed, so we only have data from the time of broadcast (29, 35). Likewise, the tools used, except in a few cases, have not allowed us to assess the degree of real impact on audience attitudes and behaviors (32, 34, 41, 44).

### Evaluation of the quality of the selected studies

**Figure 4:**
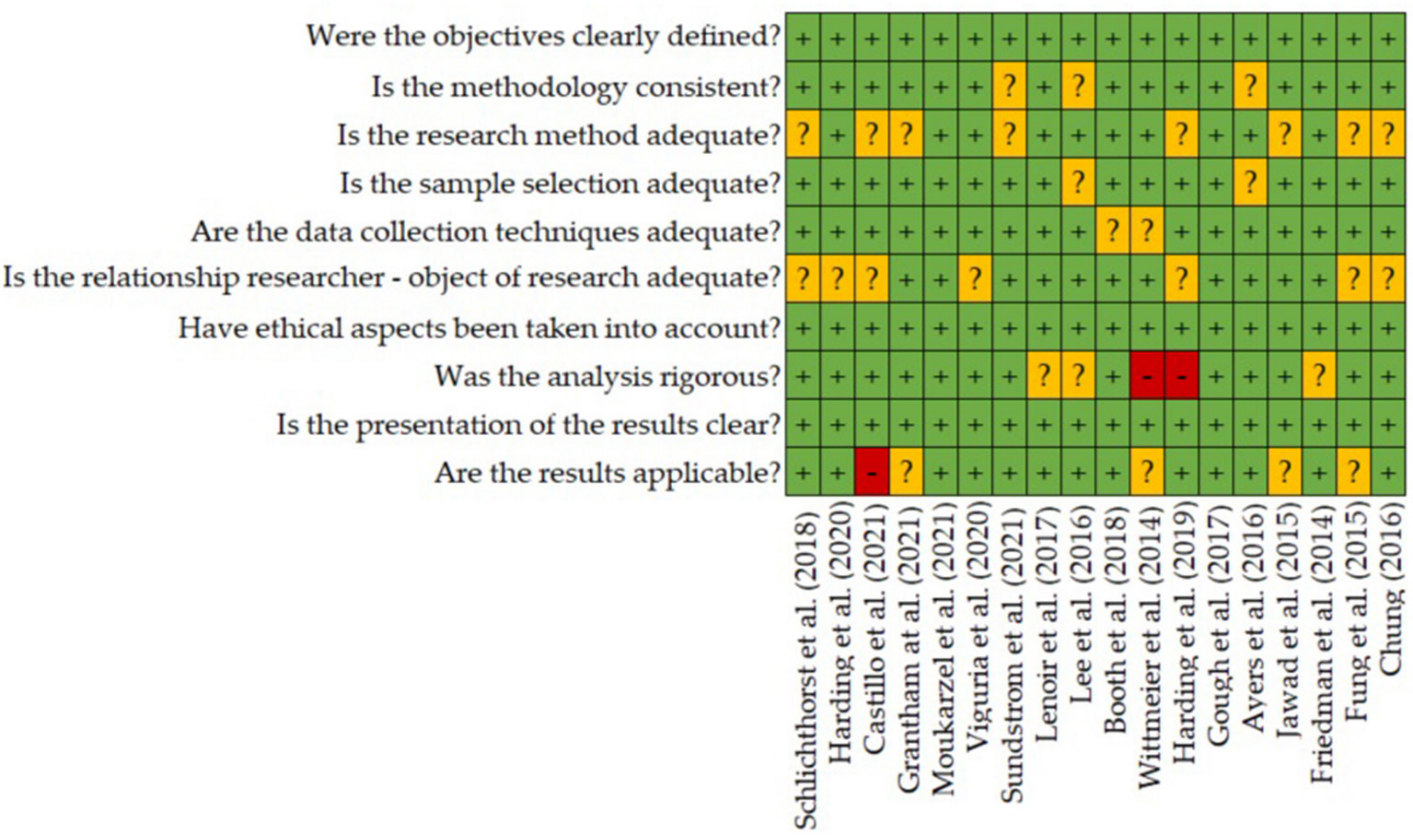
Evaluation of the quality of the selected articles using the “Critical Appraisal Skills Programme” tool.

To ensure that no selected study could interfere with or distort the conclusions of this systematic review, the Critical Appraisal Skills Programme (CASP) quality assessment tool was applied. This instrument makes it possible to assess the level of rigor, credibility and relevance of a study by means of ten questions (45). The results obtained from the evaluation of the selected articles are shown in Figure 4. All of them have a low risk of bias, so they have been included in the review. In this process, no article chosen in the selection process has been eliminated.

## Discussion

The core aim of this systematic review was to explore and analyze existing scientific studies evaluating the effectiveness of behavioral-based public health communication campaigns broadcast on Twitter. Social networks offer multiple advantages for the promotion of awareness campaigns (46). However, it is important to conduct periodic evaluations of these campaigns' actual impact on the population. This process will provide valuable information to make appropriate adjustments in future communication campaigns' design, approach or message.

However, evaluations of the impact of this type of awareness campaigns are not usually carried out. Only 18 articles were found that met the inclusion criteria. Thus, given the large number of campaigns that are based on Twitter sharing, or that use this social network as a complement to other broadcasting channels, it is surprising that there is so little research aimed at evaluating the impact of these campaigns (47). Despite this, the fact that studies of this type have been carried out in countries with very different sociocultural characteristics indicates that, in recent years, there is some interest in knowing the degree of effectiveness of these strategies.

### Why are public health campaigns broadcast on social networks not evaluated?

The scarcity of analysis of the impact of campaigns is a common problem in social marketing, regardless of the subject matter and the media used to disseminate them (48). Cost-effectiveness might be one of the reasons explaining this scarcity (49). In practice, many social campaigns are awarded to a particular agency or company because of the economic advantage of their proposal compared to the ones submitted by other candidates. As a result of such a managerial process, all resources tend to be allocated to the design and implementation of the campaign rather than effectiveness evaluations. This entails a pitfall because large amounts of material and economic resources may be allocated to campaigns that are not effective (50). Worse still, erroneous awareness strategies may be perpetuated in future campaigns because their ineffectiveness has not been detected.

There are other factors that may be influencing this situation. For example, the scientific literature evidences certain limitations in measuring the effectiveness of social campaigns (51). The main shortcoming in this regard is that the evaluation instruments adopted for this purpose evaluate perceived effectiveness by the users, assuming it is a direct antecedent of real effectiveness. This is a problematic assumption, though. Therefore, a better and more accurate alternative would be to use cognitive neuroscience tools that allow direct assessment of actual efficacy (52). However, these measurement tools usually entail unaffordable economic costs and therefore, difficult to be adopted.

These limitations are exacerbated in campaigns broadcast through social networks (53). Our review points out the methodological limitations and in the access to information existing in digital impact assessment tools (27, 29). Therefore, those responsible for the development and implementation of this type of campaigns might need to be more cautious when measuring their effectiveness, because the validity and reliability of the current tools are questionable.

### Effectiveness of Twitter campaigns: Current limitations and evaluation proposals

Added to the aforementioned, one of the main problems in resolving the issue is to identify variables to be considered to evaluate the effectiveness of a public health campaign (54). From a product marketing perspective, there is no unanimous agreement on what factors measure the impact of advertising campaigns in social networks. In this regard, Raudeliuniene et al. (55) identified 39 primary factors in the literature, including digital marketing analytics (website traffic, impressions or content relevance), sales, customer attraction, audience retention, and consumer engagement, among others.

However, the factors are inevitably different for the evaluation of social advertising (56). Thus, in the first instance, Twitter statistics could be evaluated, in the form of metrics, impressions, reach, geographic areas where most interaction occurs, and user profile. However, this information is not sufficient for two reasons. First, these are immediate results that do not take into account the subsequent evolution of the degree of effectiveness of the campaign (29, 34). Therefore, at least, it would be possible to analyze the statistics of this social network in time periods subsequent to the moment of the campaign's broadcast. This would allow a follow-up of its impact on networks, and thus better assess whether the campaign has proved effective (34). Even so, the systematic review carried out points out limitations in the access to users' information and in the recording of their messages (32, 41). For this reason, these data should be complemented with other tools that assess behavioral change.

Second, most of the research reviewed assesses impact solely using Twitter metrics (27, 30, 31, 40). Therefore, one should be restrained in the interpretation of their results because a campaign with a high digital impact, is not always synonymous with a high level of effectiveness (28). In this sense, we could go a step further and analyze the content of the interactions with the tweets and hashtags of the campaign. In this way, it would be possible to know the main topics derived from it and analyze the sentiment aroused in the audience (28–57). In this regard, Sentiment Analysis tools specially designed for social network interactions (58, 59) have proved to be a promising area worthy of further attention (60).

Additionally, from a social advertising perspective, a campaign is considered effective when it manages to raise awareness among the audience about a certain issue, promoting a change in their attitudes and behaviors. Therefore, qualitative evaluation tools could be applied, such as semi-structured interviews, focus groups or anonymous mass surveys, which would allow knowing the real change achieved through communication strategies (61, 62). These instruments should identify the degree of recall, impact, attitudes, and healthy or prevention behaviors acquired after the campaign broadcast (63, 64).

The current evidence is insufficient to support the effectiveness of Twitter as a broadcasting platform for public health campaigns in general. However, some advantages make it worthy of consideration and further studies, especially having in mind its cost-effectiveness and the broad audience they can reach, especially if compared with traditional programs or interventions based on “aged” communication sources, such as TV, radio and paper-based media (15, 18). The apparent economic benefit such platforms offer can justify them, at least, as a perfect complement for multimedia campaigns, especially to reinforce the message for younger audiences (43).

### Limitations of this systematic review and future lines of research

This systematic review was carried out following the PRISMA procedure to avoid possible biases in the selection and/or recording of data. In addition, the inclusion/exclusion criterion was that the eligible articles should form part of relevant indexes and databases worldwide to guarantee, as far as possible, the quality of the research.

Despite all this, the present systematic review is not exempt from the limitations characteristic of this type of study. Thus, the review may present publication bias. This bias occurs when research with negative” or non-significant results is either published in journals of lower impact or not published at all. Therefore, they may not have been included in the review (65).

Moreover, the final low number of original research papers that met the eligibility criteria makes it remarkable the high number of technical shortcomings and potential quality flaws found in these papers. This indeed is one of the conclusions of the conducted review, i.e., that the scientific literature on the evaluation of health campaigns issued through Twitter is really scarce. However, this circumstance could limit the breadth and scope of the research findings. Furthermore, it is possible that the non-indexed literature could have provided more interesting information on this subject (although it could have methodological limitations or limitations in the quality of the results). Also, it is worth mentioning that none of the sources found corresponded to longitudinal or time-based research, something that might be of great use to measure the successfulness and stability of the effects produced by these actions/program over time (66).

The present study has provided some guidelines for the improvement of public health campaigns. However, future studies could try to address this aspect and develop a standard and accessible tool to evaluate the impact of social communication campaigns carried out through Twitter. In this way, it could be easier to measure the effectiveness of awareness campaigns, and data of interest for the development of future communication strategies could be obtained.

## Conclusions

Contrary to what was initially hypothesized on the basis of the study background, the results of the studies analyzed in this systematic review do not provide clear evidence on the suitability of Twitter as an effective communication channel for the promotion of healthy behaviors.

On the other hand, it is noteworthy that the evaluation of the quality of the studies analyzed gave considerably positive results endorsing their key features and scientific rigor in many terms, but the adequacy of both research methods and researcher-object relationship represent frequent constraints among these scientific studies.

In addition, the scarcity of research that performs post-campaign follow-up, as well as the lack of measurement on the degree of awareness and behavioral change, are manifested. These factors limit the findings of the studies, since they do not take into account the real effectiveness of the campaign, but only its digital impact.

The usefulness of this review is fundamentally practical since it offers information of interest for the development of communication strategies on Twitter. Thus, the limitations of current evaluation tools are discussed, and more complete evaluation methodologies are proposed to measure the impact of public health campaigns on social networks.

## Data availability statement

The original contributions presented in the study are included in the article/supplementary material, further inquiries can be directed to the corresponding author/s.

## Author contributions

MF and FA conceived and designed the research and contributed with reagents, materials, and analysis tools. MF, SU, and AJ analyzed the data and wrote and revised the paper. All authors contributed to the article and approved the submitted version.

## Funding

This work was supported by the Research Grant ACIF/2020/035 (MF) from Generalitat Valenciana. Funding entities did not contribute to the study design or data collection, analysis and interpretation or writing of the manuscript.

## Conflict of interest

The authors declare that the research was conducted in the absence of any commercial or financial relationships that could be construed as a potential conflict of interest.

## Publisher's note

All claims expressed in this article are solely those of the authors and do not necessarily represent those of their affiliated organizations, or those of the publisher, the editors and the reviewers. Any product that may be evaluated in this article, or claim that may be made by its manufacturer, is not guaranteed or endorsed by the publisher.
